# 968. Universal Newborn Testing for Congenital Cytomegalovirus (cCMV) Infection Comes of Age: Clinical Sensitivity of Screening Tests and Infant Outcomes in a cCMV Screening Study in Minnesota

**DOI:** 10.1093/ofid/ofad500.2462

**Published:** 2023-11-27

**Authors:** Mark R Schleiss, Erin Osterholm, Nelmary Hernandez-Alvarado, Rebecca Kruc, Hannah Herd, Abbey Sidebottom, Sondra Rosendahl Licensed Genetic, Sheila Dollard, Tatiana Lanzieri

**Affiliations:** University of Minnesota, Minneapolis, MN; University of Minnesota Medical School, Minneapolis, Minnesota; University of Minnesota Medical School, Minneapolis, Minnesota; University of Minnesota Medical School, Minneapolis, Minnesota; University of Minnesota/MHealth Medical Center, Minneapolis, Minnesota; Allina Health Research Institute, Minneapolis, Minnesota; Minnesota Department of Health, Saint Paul, Minnesota; Centers for Disease Control and Prevention, Atlanta, Georgia; Centers for Disease Control and Prevention, Atlanta, Georgia

## Abstract

**Background:**

Universal congenital cytomegalovirus (cCMV) screening is controversial. In 2023, we completed a study evaluating analytical and clinical sensitivity of dried blood spots (DBS) for detection of cCMV infection and disease. Analytical sensitivity is the ability of an assay to detect infection, while clinical sensitivity is an assay's ability to identify individuals with disease. The completion of this study coincided with implementation of new legislation making Minnesota the first state in the USA to mandate universal cCMV testing.

**Methods:**

In six newborn nurseries in Minnesota, consented newborns were screened for cCMV, comparing PCR of saliva with PCR of the DBS obtained for routine newborn screening. Screen-positive infants had a confirmatory urine PCR, and a diagnostic clinical evaluation. Using international consensus criteria, we categorized cCMV cases as: moderate-to-severe; mildly symptomatic; asymptomatic with isolated sensorineural hearing loss (SNHL); or asymptomatic.

**Results:**

From February 2016 - January 2023, among 23,644 newborns screened, 87 (3.7 per 1,000) were identified with cCMV. Analytical sensitivity of saliva was 93.1% (81); for DBS, it was 73.6% (64) by the UMN lab, and 77.0% (67) by the CDC lab. At birth, 67 (77%) infants were asymptomatic; 6 moderately-to-severely symptomatic (2 with SNHL); 10 mildly symptomatic; and 4 asymptomatic with isolated SNHL. 4 infants had delayed-onset SNHL (2 asymptomatic, 2 symptomatic). Among 20 infants with symptomatic cCMV at birth and/or SNHL, clinical sensitivity of saliva was 95% (19), and for DBS was 75% (15) in the UMN lab, and 85% (17) in the CDC lab.

**Conclusion:**

This unselected screening study demonstrated a prevalence of 3.7/1000 of cCMV in Minnesota newborns. The enhancement in clinical sensitivity of DBS (over analytical sensitivity) to identify children with symptoms or sequelae suggests their usefulness for cCMV screening, though most identified infants will be asymptomatic. In parallel with this work, in the spring of 2023, Minnesota became the first state to screen all newborns for cCMV, using the newborn DBS. Continued evaluation of CMV DBS testing methods for reproducibility, efficiency, high-throughput capability and clinical correlation will be important as other states consider universal cCMV screening.

**Disclosures:**

**All Authors**: No reported disclosures

